# A novel apolipoprotein E mutation, ApoE Ganzhou (Arg43Cys), in a Chinese son and his father with lipoprotein glomerulopathy: two case reports

**DOI:** 10.1186/s13256-022-03302-0

**Published:** 2022-02-23

**Authors:** Runxiu Wang, Chengbo Zhao, Wen Chen, Zhiping Liu, Fuhua Xie

**Affiliations:** 1 The first Affiliated Hospital, Gannan Medical University, Ganzhou, 341000 Jiangxi China; 2 School of Basic Medicine, Gannan Medical University, Ganzhou, 341000 Jiangxi China

**Keywords:** Case report, Apolipoprotein E, Lipoprotein glomerulopathy, ApoE Ganzhou

## Abstract

**Background:**

Lipoprotein glomerulopathy is a rare and newly recognized glomerular disease that can lead to kidney failure. Its pathological features include the presence of lipoprotein embolus in the loop cavity of glomerular capillaries. It is believed that apolipoprotein E gene mutation is the initiator of the disease. Since the discovery of lipoprotein glomerulopathy, 16 different apolipoprotein E mutations have been reported worldwide, but most of these cases are sporadic. Here we report two cases of lipoprotein glomerulopathy, a Chinese son and his father, with a novel apolipoprotein E mutation, ApoE Ganzhou (Arg43Cys).

**Case presentation:**

Case 1, a 33-year-old Chinese man, was hospitalized on 3 March 2014 owing to edema and weakness of facial and lower limbs for 1 month. Laboratory data showed urine protein 3+, hematuria 2+, serum creatinine 203 μmol/L, uric acid 670 μmol/L, total cholesterol 12.91 mmol/L, triglyceride 5.61 mmol/L, high-density lipoprotein 1.3 mmol/L, low-density lipoprotein 7.24 mmol/L, apolipoprotein B 2.48 g/L, and lipid protein (a) 571 mg/L. Renal tissue examined by immunofluorescence and electron microscopy indicated lipoprotein glomerulopathy. Case 2, 55-year-old father of case 1, was hospitalized on 12 January 2016 owing to edema of his lower extremities for 6 months. Laboratory data showed urine protein 2+, hematuria 2+, serum creatinine 95 μmol/L, uric acid 440 μmol/L, total cholesterol 4.97 mmol/L, triglyceride 1.91 mmol/L, high-density lipoprotein 1.18 mmol/L, low-density lipoprotein 3.12 mmol/L, apolipoprotein B 2.48 g/L, and lipid protein (a) 196 mg/L. Renal tissue examined by immunofluorescence and electron microscopy indicated lipoprotein glomerulopathy. Apolipoprotein E mutation test showed that they had the same gene mutation, a novel type of apolipoprotein E mutation.

Based on their clinical presentation and examination findings, they were diagnosed with lipoprotein glomerulopathy. Case 1 was treated with prednisone and dual plasma replacement, followed by simvastatin, nifedipine, triptolide, and angiotensin II receptor blocker drug therapy. After 1 month, the edema symptoms of the patient were alleviated, and urinary protein, serum creatinine, and uric acid were quantitatively reduced. Case 2 was treated with *Tripterygium wilfordii* and angiotensin II receptor blocker drugs for 3 weeks, and his edema symptoms were alleviated, and urinary protein, serum creatinine, and uric acid were quantitatively reduced.

**Conclusions:**

The apolipoprotein E mutation in the two cases we reported was a familial aggregation phenomenon, and the mutation is a novel type, which we named ApoE Ganzhou (Arg43Cys). The location of the gene mutation is close to the most common mutation type of lipoprotein glomerulopathy, ApoE Kyoto (Arg25Cys), so we speculate that its pathogenic role might be the similar to that of ApoE Kyoto (Arg25Cys).

## Background

Lipoprotein glomerulopathy (LPG) is a new type of glomerular disease characterized by lipid deposition in the glomeruli. As early as 1987, renal lipidosis was described in a review by Faraggiana *et al*., but was not considered an independent glomerular disease at the time [1]. In the same year, Saito *et al*. reported this disease in the 17th Annual Meeting of the Japan Society of Nephrology, and 2 years later, the disease was determined to be an independent disease and named LPG [2]. Since the discovery of LPG, 16 different apolipoprotein E (ApoE) mutations have been reported worldwide [3], most of which were from East Asian countries such as Japan [4–6] and China [7, 8].There were also a few reports on ApoE mutations of LPG from European [9–13] and American [14–16] countries. However, the exact pathogenesis of the disease has not been fully elucidated.

Here, we report two patients with LPG, a Chinese son and his father, with a novel apolipoprotein E mutation, ApoE Ganzhou (Arg43Cys).

## Case presentation

### Case 1

A 29-year-old Chinese male was admitted to the Department of Nephrology, the First Affiliated Hospital of Gannan Medical University for edema of face and lower limbs. Physical examination results showed that he had 167 cm body length, 54.5 kg body weight, and 184/110 mmHg blood pressure. He had slightly swollen eyelids, no xanthoma, no Achilles tendon thickening, no abnormalities in heart, lung, or abdomen, and mild edema of lower limbs.

**Fig. 1.**
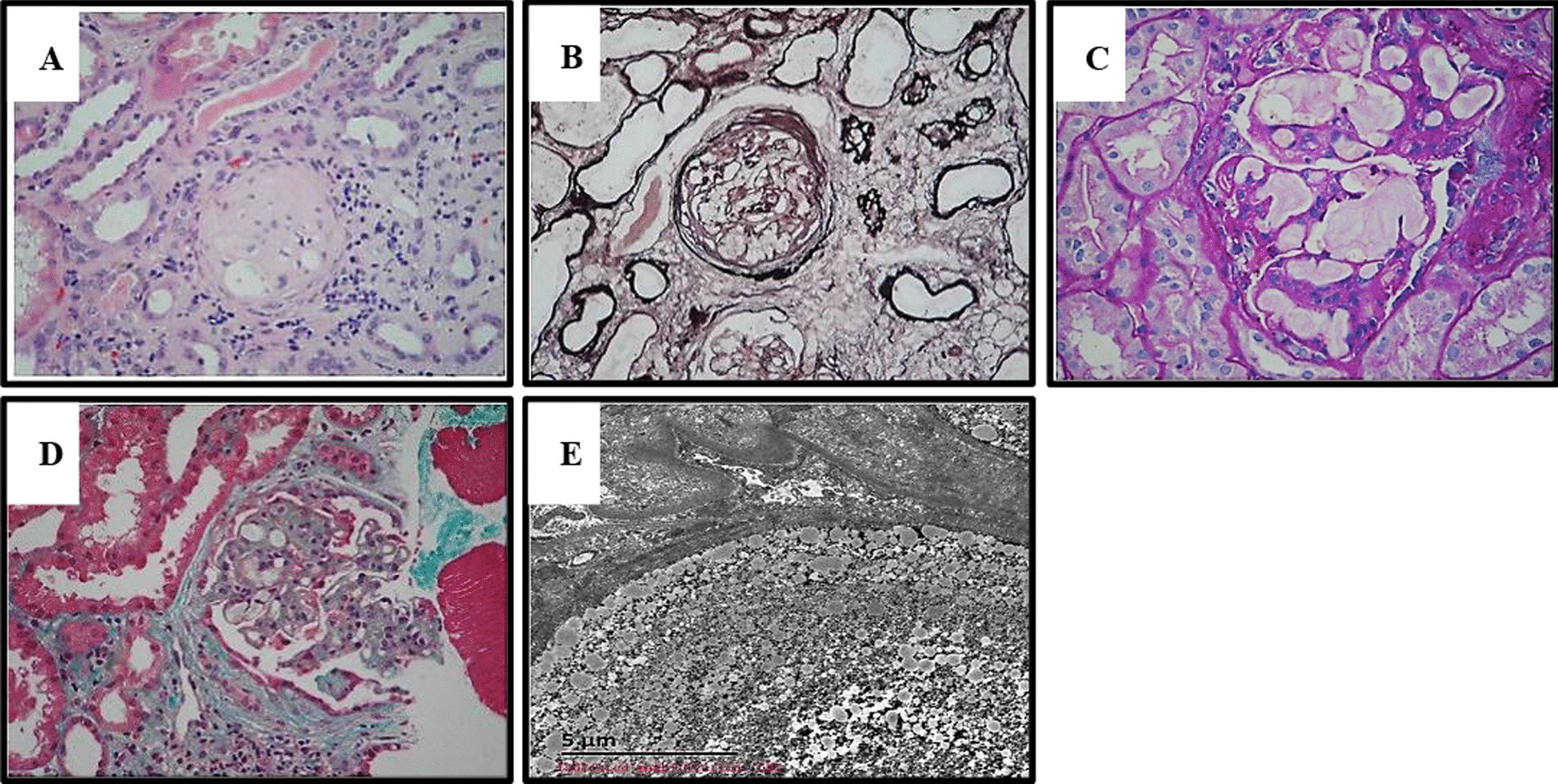
Histological analysis for the renal biopsy specimens in case 1 under light microscope. **A** Hardened glomerulus [hematoxylin and eosin (HE) staining, ×200]. **B** Glomerular periballoon fibrosis [periodic acid–Schiff–methenamine (PASM) staining, ×200]. **C** Dilation of capillary loops and presence of thrombotic substances in the lumen [periodic acid–Schiff (PAS) staining, ×200]. **D** Polyhemoglobin deposition in the glomeruli (Masson staining, ×200). **E** Electron microscopy results showing capillary endothelial cells with obvious vacuolar degeneration and a large number of lipid vacuolar protein substances in the cavity (scale bar, 5 μm)

Laboratory examination results are presented in Table 1. After admission, he was orally given prednisone with dosage of 55 mg/day. One week later, a renal biopsy was collected and 17 glomeruli were examined under light microscope. It was found that one glomeruli was hardened, the rest were enlarged, and the capillary loops were highly dilated (Fig. 1A). Fibrosis around glomerular balloon was also observed (Fig. 1B). There was dilation of capillary loops, and thrombotic substances were found in the lumen (Fig. 1C). There was no obvious polyhemoglobin deposition in the glomeruli (Fig. 1D). Immunofluorescence microscopy showed that immunoglobulin A (IgA) was positive (not shown). Electron microscopy results showed capillary endothelial cells with obvious vacuolar degeneration and a large number of lipid vacuolar protein substances in the cavity (Fig. 1E).

**Table 1:** Results of patients’ laboratory examination

Patient	Urinalysis	RFT	LFT	Lipid profile
Case 1	UP 3+ hematuria 2+	Scr 203 μmol/L UA 670 μmol/L	ALT 12 U/L AST 16 U/L A 22.8 g/L	TC 12.91 mmol/L TG 5.61 mmol/L HDL 1.3 mmol/L LDL 7.24 mmol/L ApoB 2.48 g/L LP 571 mg/L
Case 2	UP 2+ hematuria 2+	Scr 95 μmol/L UA 440 μmol/L	ALT 21 U/L AST 20 U/L A 24.9 g/L	TC 4.97 mmol/L TG 1.91 mmol/L HDL 1.18 mmol/L LDL 3.12 mmol/L ApoB 2.48 g/L LP 196 mg/L

*UP* urine protein, *RFT* renal function test, *Scr* serum creatinine, *UA* uric acid, *ALT* alanine transaminase, *AST* aspartate transaminase, *A* albumin, *LFT* live function test, *TC* total cholesterol, *TG* triglyceride, *HDL* high-density lipoprotein, *LDL* low-density lipoprotein, *ApoB* apolipoprotein B, *LP* lipid protein

Based on the above clinical manifestations and examination results, the patient was diagnosed with LPG. The dose of prednisone was rapidly reduced to 15 mg for oral maintenance in a short period of time. Meanwhile, double plasma replacement was performed, and simvastatin lipid-lowering drug triptolide (60 mg/day) and blood-pressure-lowering drug angiotensin II receptor blocker (ARB) were administered. The patient has been receiving dialysis treatment in our hospital since discharge.

**Fig. 2:**
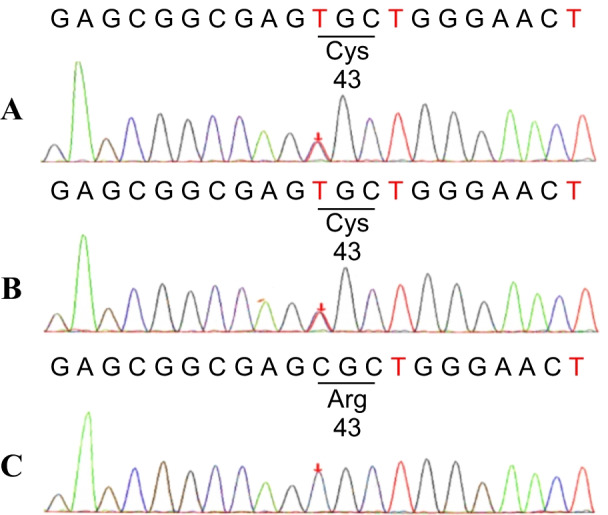
DNA sequence analysis for ApoE mutation. ApoE gene was sequenced in genomic DNA from both patients (**A**, case 1; **B**, case 2) and a family member (**C**, mother of case 1). Both patients had a heterozygous ApoE mutation of C-to-T transition in exon 3 that changed the amino acid at position 43 of the mature protein from arginine to cysteine. Cys, cysteine; Arg, arginine

**Fig. 3:**
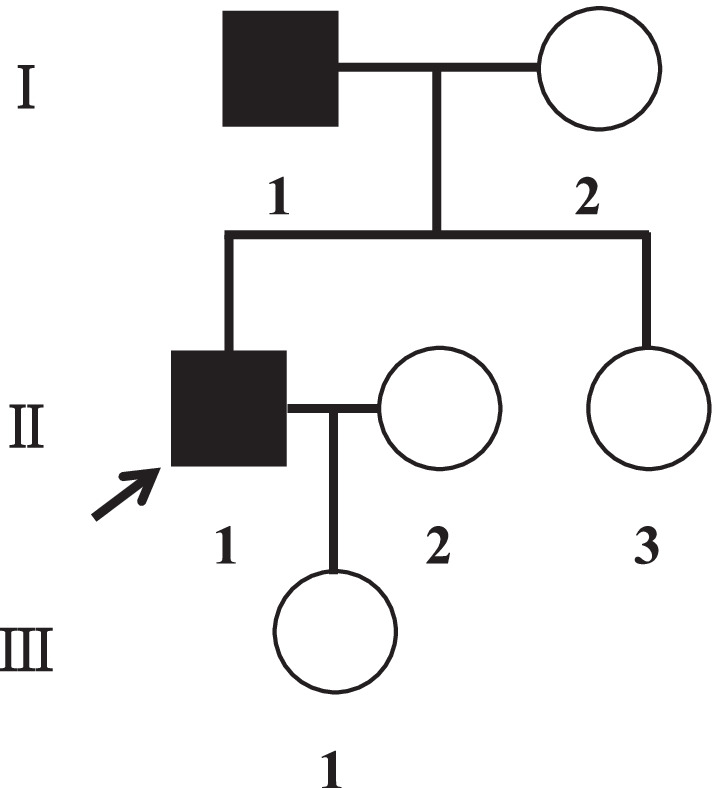
Pedigree of the family. The proband (II-1, case 1) in this case report is indicated by the arrow. The square and the circle represent male and female, respectively. Blank and black symbols respectively represent unaffected family members and patients for ApoE Ganzhou (Arg43Cys). The gene mutation of the proband came from his father, I-1, case 2 in this report. Cys, cysteine; Arg, arginine

We did not perform ApoE gene mutation test for this patient at that time. About 2 years later when his father was diagnosed with LPG, we decided to examine genetic factors for these two individuals. Kidney genomic DNA was collected from these two and sequenced. The results showed that nucleotide 127 of the ApoE-gene-encoding region was mutated from cytosine to thymine (g.127C>T), which resulted in a missense mutation of amino acid 43 from arginine to cysteine (Fig. 2). His father had the same ApoE mutation, while his mother had no mutation. This is a novel mutation in ApoE gene, and we named it ApoE Ganzhou (Arg43Cys). We searched the GnomAD database for this DNA sequence variant, and found that this mutation was most common in East Asia and Europe (Finland), with frequencies of 0.00005445 and 0.00004654, respectively. We investigated his family history and found that his mother, sister, and daughter had no symptoms of LPG (Fig. 3). Therefore, he obtained the gene mutation from his father, and the disease is an autosomal dominant genetic disorder.

### Case 2

A 51-year-old Chinese male, father of case 1, was admitted to the Department of Nephrology, the First Affiliated Hospital of Gannan Medical University for edema of lower limbs approximately 2 years after his son was diagnosed with LPG. Physical examination showed that he had 167 cm body length, 56.5 kg body weight, and 139/88 mmHg blood pressure. He had no edema in the face, no abnormal heart, clear breathing sound but audible moist rales in both lungs, no abnormal abdomen, and no percussion pain in the renal area. He had slight edema of lower limbs.

**Fig. 4.**
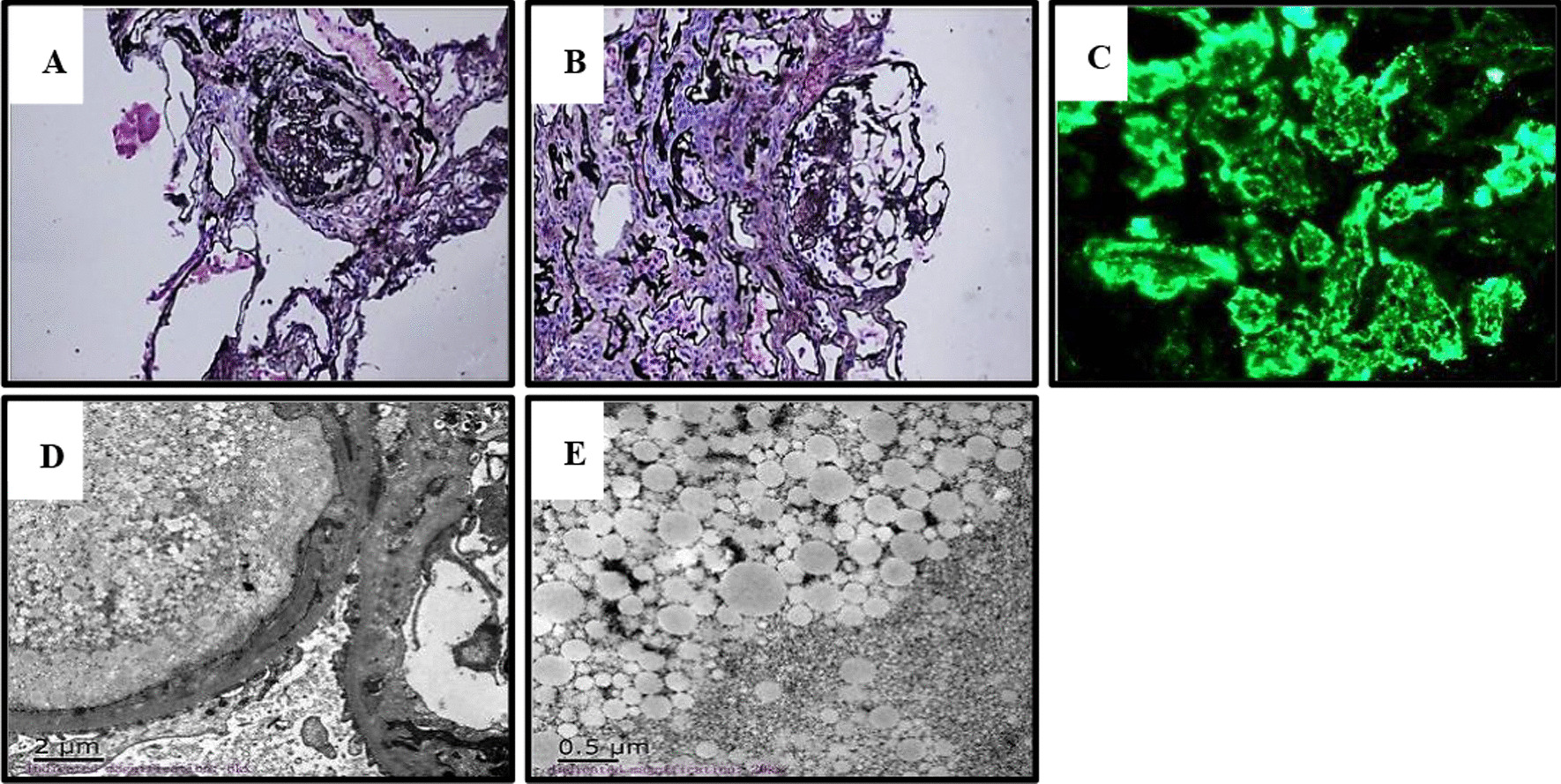
Histological analysis of the renal biopsy specimens in case 2. **A** Hardened glomerulus under light microscope (PASM staining, ×200). **B** Glomerulus with segmental sclerosis under light microscope (PASM staining, ×200). **C** Immunofluorescence microscopy results showing positive ApoE. **D** and **E** Electron microscopy results showing capillary endothelial cells with obvious vacuolar degeneration, and a large number of lipid vacuolar protein substances in the cavity (scale bar, 2 μm for **D** and 0.5 μm for **E**)

Laboratory examination results are presented in Table 1. Renal biopsy revealed four glomerular scleroses (Fig. 4A) and one glomerular segmental sclerosis (Fig. 4B). It was found that the capillary loops of the rest of the glomerulus without sclerosis were highly dilated, and the cavities were filled with lightly stained, vacuolated thrombotic substances. Oil Red O staining results of kidney were positive (data not shown). Immunofluorescence microscopy showed that ApoE was positive (Fig. 4C). Electron microscopy showed that capillary endothelial cells were obviously degenerated (Fig. 4D), and a large number of lipid vacuolar protein substances were found in the cavity (Fig. 4E). ApoE gene sequencing revealed that he had the same mutation, ApoE Ganzhou (Arg43Cys), as his son (Case 1) (Fig. 2).

According to the clinical manifestations and examination results, the patient was diagnosed with LPG and given *Tripterygium wilfordii* (60 mg/day), ARB drugs, and other treatments. His condition is stable at present.

### Follow-up and outcomes

Case 1 developed end-stage renal disease (ESRD) 4 years after discharge and underwent maintenance hemodialysis until now. Case 2 was followed up for 4 years and his renal function is stable at present.

## Discussion and conclusions

**Fig. 5:**
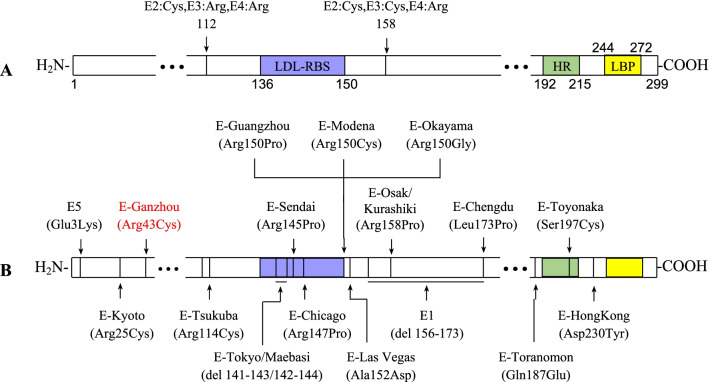
Structure and mutations of ApoE. **A** Important regions of ApoE and the difference among E2, E3, and E4 at positions 112 and 158. **B** Seventeen different ApoE mutations reported, and a novel mutation, ApoE Ganzhou (Arg43Cys), marked in red. *LRBS* LDL-receptor binding site, *HR* hinge region, *LBP* lipid binding position, *Cys* cysteine, *Arg* arginine, *Glu* glutamic acid, *Lys* lysine, *Pro* proline, *Gly* glycine, *Leu* leucine, *Ser* serine, *del* deletion, *Ala* alanine, *Gln* glutamine, *Asp* aspartic acid, *Tyr* tyrosine

The exact pathogenesis of LPG has not been fully elucidated, but ApoE gene mutation was recognized as one of causes of the disease. ApoE is a glycoprotein whose mature form consists of 299 amino acid residues (molecular weight ~ 34 kD) (Fig. 5A). ApoE contains several primary domains, including LDL-receptor binding site (LRBS, amino acids 136–150), hinge site (HR, amino acids 192–215), and lipid binding region (LBR, amino acids 244 to 272) [3]. ApoE is mainly composed of HDL and very-low-density lipoprotein (VLDL). By binding with LDL or LDL-related protein, it mediates the uptake of lipoprotein (mainly triglyceride) by cells, and plays an important role in the metabolism of blood lipid. The genotypes of ApoE are 2, 3, and 4, and their corresponding monomer phenotypes are E2, E3, and E4, respectively. The difference between them lies in the distinction of amino acid residues at positions 112 and 158. Both are cysteine in E2, and arginine in E4, but arginine and cysteine locate in E3. Human ApoE consists of two monomer phenotypes, and the most common phenotype is ApoE3/3.

Various ApoE gene mutations have been found in patients with LPG. The first reported mutation was ApoE Sendai (Arg145Pro) [2]. So far, 16 different types of gene mutations have been reported worldwide (Fig. 5B). Nearly half of mutations occurred in the LDL-receptor binding site (LRBS), such as ApoE Sendai (Arg145Pro) [2], ApoE Chicago (Arg147Cys) [16], ApoE Guangzhou (Arg150Pro)[17], ApoE Modena (Arg 150Cys) [11], ApoE Okayama (Arg150Gly) [18], ApoE Tokyo (Del 141-143) [19], and ApoE Maebasi (Del 142-144). These mutations might result in reduced binding capacity of ApoE protein to LDL receptor. Other mutations locate outside of LRBS, such as ApoE5 (Glu3Lys) [20], ApoE Kyoto (Arg25Cys) [21], ApoE Tsukuba (Arg114 Cys) [22], ApoE Las Vegas (Ala152Asp) [14], ApoE1 (Del 156-173) [23], ApoE Osak/Kurashiki (Arg158 Pro) [24], ApoE Toranomon (Gln187Glu) [25], ApoE Toyonaka (Ser 197Cys) [26], and ApoE Hong Kong (Asp230Tyr) [27]. These mutations may change the spatial conformation and stability of ApoE protein.

LPG has a wide range of age distribution, and occurs in all patients aged 4–69 years old. However, recent cases have also been reported in newborn baby and people in their seventies [28]. The incidence of LPG is approximately 2:1 in men and women. In previous literature reports, most cases were sporadic, and some cases were familial [7, 8, 15, 29–33]. Patients presented with varying degrees of proteinuria, mostly with nephrotic syndrome, and a few with only mild proteinuria. About one-half of patients developed end-stage renal disease with varying durations. Some patients presented with hypertension, arteriosclerosis, liver function abnormality, and other systemic manifestations, but the degree was very light. Most patients with LPG had abnormal lipoprotein and dyslipidemia, similar to type III hyperlipoproteinemia [3]. Histological features are the most important evidence for the diagnosis of LPG [2]. Under light microscopy, the capillary lumen is typically highly dilated and filled with lightly stained reticular material. Mesangial dissolution and light to moderate mesangial hyperplasia are often seen. Some mesangial matrix may be inserted into the basement membrane of the glomeruli, forming a double orbital sign. Oil Red O staining shows the presence of lipid droplets in the capillary lumen. Immunofluorescence microscopy shows positive staining of ApoE and ApoB in lumen thrombotic substances. The capillary cavity is filled with lipid vacuoles under electron microscope.

At present, there is no specific treatment for LPG, and adrenal corticosteroids, cytotoxic drugs, and anticoagulants have no obvious effect. Lipid-lowering therapy has been reported to relieve hyperlipidemia and proteinuria [2], while plasmapheresis or LDL removal therapy has also been reported to reduce proteinuria and proteinuria deposition, but the effect on long-term prognosis of patients is unknown.

In summary, the ApoE mutation in the two patients we reported was a familial aggregation phenomenon, and the mutation is a novel type, which we named ApoE Ganzhou (Arg25Cys). SIFT and PolyPhen were used to predict the effect of the mutation, and the predicted scores were 0.008 and 0.999, respectively, indicating that the mutation was harmful to the function of ApoE protein. Also, the location of the gene mutation is close to the most common mutation type of LPG, ApoE Kyoto (Arg25Cys), so we speculate that its pathogenic role might be the similar to that of ApoE Kyoto (Arg25Cys). From the medical history, the son (case 1) had earlier onset, presenting as nephrotic syndrome, poor response to hormone and immunosuppressive therapy, rapid disease progression (entering end-stage renal disease in 3 years), and concurrent renal replacement therapy. However, the onset time of the father (case 2) was late, and the disease was relatively mild. The father was followed up for 4 years, and the renal function is stable at present. Although the proband’s sister and daughter are currently asymptomatic, we cannot rule out that they carry the mutant gene because they have refused to undergo genetic testing. However, given the wide distribution in age of patients who have been diagnosed with LPG, we urge them to come to the hospital regularly for physical check-ups.

## Data Availability

The datasets used and analyzed during the current case report are available from the corresponding author on reasonable request.

## Abbreviations

LPG: Lipoprotein glomerulopathy
Apo E: Apolipoprotein E
LRBS: LDL-receptor binding site
HR: Hinge site
ESRD: End-stage renal disease
UP: Urine protein
RFT: Renal function test
Scr: Serum creatinine
UA: Uric acid
ALT: Alanine transaminase
AST: Aspartate transaminase
A: Albumin
LFT: Live function test
TC: Total cholesterol
TG: Triglyceride
HDL: High-density lipoprotein
LDL: Low-density lipoprotein
ApoB: Apolipoprotein B
LP: Lipid protein
ARB: Angiotensin II receptor blocker
